# The Effect of Chitin Size, Shape, Source and Purification Method on Immune Recognition

**DOI:** 10.3390/molecules19044433

**Published:** 2014-04-10

**Authors:** Francisco J. Alvarez

**Affiliations:** National Center for Geriatrics and Gerontology Research Institute, Obu, Aichi 474-8511, Japan; E-Mail: alvarez@ncgg.go.jp; Tel.: +81-801-5978-018

**Keywords:** chitin, *Candida albicans*, *Aspergillus fumigatus*, *Mucor circinelloides*, PBMCs, fungal cell wall, small chitin particles

## Abstract

The animal immune response to chitin is not well understood and needs to be investigated further. However, this is a challenging topic to study because of the technical difficulties in purifying chitin, and because this material usually comes associated with contaminating components that can activate the immune system. In this study, improvements to previously described purification protocols were investigated for chitin obtained from different sources, including commercial shellfish, *Candida albicans* yeast and hyphal cell walls, as well as cell walls of the filamentous fungi *Aspergillus fumigatus* and *Mucor* *circinelloides*. The immune response to these different chitin preparations was tested using human peripheral blood mononuclear cells. In agreement with previous literature, small chitin particles of an average size of 0.2 µm were not immunogenic. On the other hand, bigger chitin particles induced in some cases a pro-inflammatory response. The results of this work suggest that not only the purity and size of the chitin particles, but also their shape can influence immune recognition.

## 1. Introduction

Chitin is a polymer of β-1,4-*N*-Acetylglucosamine (GlcNAc) that is very abundant in the animal world, although it is not synthesized by humans. It is mostly known as a component of the exoskeleton of arthropods and the cell walls of fungi [1], but it is also common in cephalopods and has been found in one species of fish [2]. In nature chitin chains can assemble into larger bundles in at least three different manners that have been termed α, β and γ chitin. α-Chitin is composed of antiparallel chains of GlcNAc, allowing for strong intermolecular bonding. It is commonly found in the shells of crustaceans like shrimp and crabs, and also in fungi. β-Chitin, which is commonly found in squid pens, contains chains aligned in a parallel fashion, which results in weaker intermolecular interactions. Finally, γ-chitin has two chains going in the same direction while a third chain goes antiparallel to them. The γ form is typical of insects and the stomach of the *Loligo* squid [3,4]. In recent years there has been an increase in research on chitin due to its potential use in biomedicine (tissue regeneration, wound healing, drug delivery, gene therapy, immunology), antimicrobial properties (food technology, agriculture) and engineering of biomaterials, among others (reviewed in [5]).

The immunogenic properties of chitin are currently an important but controversial topic. Most studies correlate exposure of animal cells to chitin with a pro-inflammatory immune response [6,7,8,9,10,11,12,13,14,15,16,17,18,19,20,21], although a few reports have shown immunosuppressant effects [22,23,24]. Some of the studies have concluded that the size of the chitin particles is important for the type of response elicited by the immune system: big particles (>40 µm) were reported to induce a classical Th2 “allergic” response, while small particles (1–10 µm) induced both protective Th1 and anti-inflammatory responses [8,24]. Particle sizes below or above those ranges do not appear to be immunogenic [24]. To date, most studies have made use of commercial shellfish chitin, and there is little information on the immunogenic properties of fungal chitin. Fungal chitin is complicated in that it is an integral part of the cell wall that is covalently linked to glucans and glycosylated proteins that can elicit strong innate immune responses (reviewed in [25,26]). Hence, specific chemical treatments are needed to purify fungal chitin away from those components.

The present study deals with the extent to which purity and size of fungal chitin could have a role in its recognition by the human innate immune system. As part of this work, improved methods were developed for the purification of chitin particles from different biological sources including commercial shellfish chitin, and the opportunistic fungal pathogens *Candida albicans*, *Aspergillus fumigatus* and *Mucor circinelloides*. The purified forms of chitin were then used to examine the immune response they elicited when co-incubated with human peripheral blood mononuclear cells (hPBMCs). This work intends to bring new light to the current debate on the immunogenic properties of purified chitin particles.

## 2. Results and Discussion

### 2.1. Purification of Commercial Chitin Results in Two Populations of Particles Very Different in Size and Homogeneity

Commercial chitin is a yellowish minced material obtained from marine food production waste, usually from shellfish like crabs or shrimps. It is composed of α-chitin, which consists of antiparallel chains of *N*-Acetylglucosamine (GlcNAc) that results in strong intermolecular bonding. The purification process from shellfish waste includes chemical treatments like deproteination with hot alkali (1 N NaOH at 65–100 °C for 1–72 h), demineralization with acid to eliminate calcium carbonate (0.275–2 M HCl at 0–100 °C for 1–48 h) and decoloration to remove pigments [27,28]. In most immunology studies it is used directly without further chemical purification, although in some labs it is homogenized by sonication and filtration through a small metal mesh [11,12,24]. In this work we first wanted to explore if further purification steps could result in chitin of a better quality for use in immunology studies.

**Figure 1:**
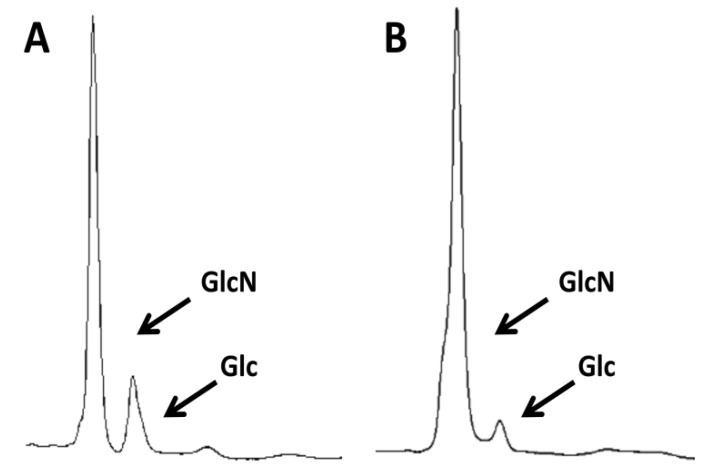
*Purity of commercial crab chitin before and after treatment under alkali and acid conditions.* Analysis of (**A**) untreated commercial crab chitin or (**B**) the same chitin that was treated by boiling twice with 5% KOH and then three times in a mix of acetic acid and H_2_O_2_. Samples were then hydrolyzed with TFAA and then the carbohydrate content was analyzed by HPLC. GlcN indicates glucosamine that results from the deacetylation of the GlcNAc under the hydrolysis conditions. Glc indicates the presence of glucose from contaminating glucans, which were not completely eliminated by the chemical treatments.

HPLC analysis of the carbohydrate content of samples of commercial crab chitin showed that they contained mostly glucosamine, as expected (the analysis deacetylates GlcNAc to GlcN). However, the level of glucans was significant (10%–17%, Figure 1A). To achieve further purification, the chitin was subjected to boiling for one hour under alkaline conditions two times and under acid conditions three times. For the alkali boils, the chitin was suspended in 5% KOH. For the acid boils the resulting pellets were suspended in a 1:1 mix of glacial acetic acid and 40% hydrogen peroxide, avoiding the usual autoclaving step typical of many previously described protocols for the purification of fungal chitin [29,30]. Surprisingly, these multiple treatments reduced, but did not completely remove, the glucan fraction (Figure 1B).

**Figure 2:**
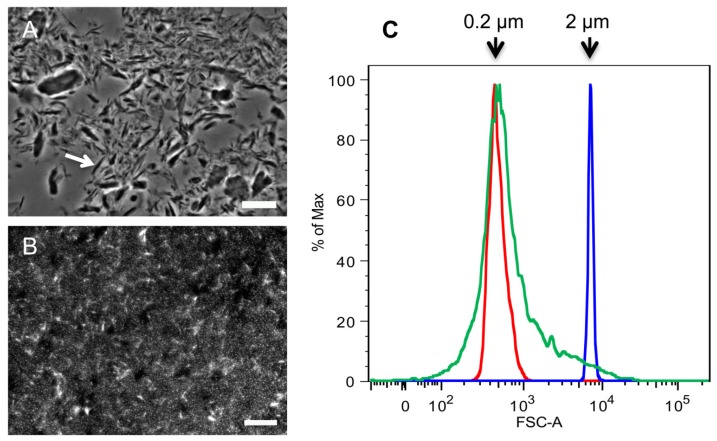
Comparison of SSCs and 2 k particles obtained from commercial crab chitin*.* Microscopic analysis of (**A**) 2 k chitin viewed by phase contrast microscopy; and (**B**) super small chitin particles (SSCs) that were stained with CFW and viewed by fluorescence microscopy; (**C**) Flow Cytometry analysis of SSCs. Red and blue indicate the profiles of 0.2 and 2 µm standard beads, respectively; green indicates the profile of SSCs. Arrow in (**A**) points to a spike-shaped chitin particle. Bars, 10 µm.

At this point the samples were not subjected to further alkali or acid treatments, and instead more attention was paid to the late washing steps. When washing chitin in a tabletop centrifuge, a pellet fraction containing white material of loose consistency was obtained. Since the spins were performed at 2057 × *g*, the end product was named “2 k” chitin. Microscopic analysis showed that the 2 k chitin was heterogeneous. Most very small particles looked like spots, others were in the shape of spikes of up to 10 µm length, and there were also bigger fragments of irregular shapes and sizes, mostly in the range of 10 to 40 µm (Figure 2A and flow cytometry data not shown).

The supernatant fractions left over from the preparation of 2 k chitin were then spun at 15,557 *×g* in a tabletop centrifuge, resulting in a white pellet. Unlike the 2 k chitin, this material behaved as a colloidal suspension that never sedimented at 1 *×g*. Fluorescence microscopy after calcofluor white (CFW) staining and flow cytometry demonstrated that the particles in this fraction had an average size of 0.2 µm and contained both round shaped particles and 1–2 µm long rods (Figure 2B,C). Since other labs had previously called any chitin fraction of less than 2 µm “super small chitin particles” or “SSCs”, this terminology was also adopted. As described below, this purification approach was also applied to the fractionation of chitin of fungal origin.

### 2.2. Differential Immune Response to 2 k Chitin and SSCs Purified from Commercial Crab Chitin

As mentioned above, there are increasing reports in the literature of chitin inducing immune responses, and there is currently a debate as to whether these responses are mainly pro- or anti-inflammatory. To examine this, the ability of representative chitin preparations to induce cytokine production by hPBMCs was measured. In these experiments chitin was used at a final concentration of 10 µg/mL, based on the signals obtained by HPLC analysis (expanded in the Experimental Section). Independent replicates were obtained by incubating each chitin batch with hPBMCs originating from different donors. ELISA were performed to detect the common pro-inflammatory cytokines IL-6, TNFα and IL-1β and the anti-inflammatory IL-10.

In agreement with previous literature [24], the SSCs from commercial crab chitin did not induce a cytokine response, while chitin from the 2 k fraction significantly induced all the pro-inflammatory cytokines tested, especially IL-6. The anti-inflammatory IL-10 did not reach significance (see Table 1), although it was close to being significant.

**Table 1:** Cytokines induced by exposure of crab chitin to hPBMCs.

Cytokine		SSCs (n = 20)	2 k chitin (n = 9)
IL-6	control	96 (80)	61 (49)
	chitin	270 (540)	2322 (1495)
	*p*	0.173	0.0019
IL-1β	control	29 (24)	40 (0)
	chitin	28 (28)	1543 (1200)
	*p*	0.914	0.01
TNFα	control	36 (7)	15 (10)
	chitin	36 (14)	78 (88)
	*p*	0.972	0.02
IL-10	control	8 (1)	56 (79)
	chitin	9 (5)	629 (577)
	*p*	0.798	0.06

Chitin preparation [number of independent replicates]; Mean (standard deviation); Values are given in pg/mL.

Since the 2 k fraction of purified chitin that induced an immune response had such a wide range of sizes and shapes, it was difficult at this point to determine what factor was more relevant for the immune recognition. Previous reports by others concluded that particles of 70–100 µm were unable to induce cytokines [24]. This meant that either the spike-like structures of up to 10 µm long or the irregularly shaped particles of various sizes could account for the observed cytokine induction. Further work described below with chitin of fungal origin was also initiated, to determine whether a similar purification protocol could be used and whether there would be any similarities with the above results for crab chitin.

### 2.3. 2 k Chitin and SSCs from C. albicans Yeast Cells Show Significant Differences with Commercial Chitin

*C. albicans* is an opportunistic fungal pathogen that lives usually as a commensal in the gut and mucosal surfaces of the human body. It is a dimorphic fungi that grows most commonly as yeasts (blastopores) or hyphae (filaments) (see Figure S1 A,B). Its cell wall contains about 40% of β-1,3 glucan, 2%–4% chitin and the rest consists of various glycosylated proteins attached through GPI anchors to β-1,6 glucans. The cell wall has both rigidity and plasticity since it changes to adapt to very diverse environmental changes, such as external pH, osmolarity or nutrient availability. As in the case of shellfish, the cell walls of fungi contain α-chitin, composed of antiparallel chains of GlcNAc. It is present all throughout the cell wall, but it appears more concentrated in a layer closest to the plasma membrane. The level of chitin has been shown to increase as a compensatory response to the defects in other cell wall components such as a reduced β-1,3-glucan synthesis caused by mutations [31] or antifungal drugs [32] and mutations affecting the glycosylation of cell wall proteins [29].

In the present work there was a specific interest in reporting the human immune responses to fungal chitin, since this is an understudied area of research. Exposure of whole *C. albicans* cells to hPBMCs results in the induction of a protective Th17 response [33]. Both IL-1β and IL-6 are two cytokines directly involved in the initiation of such response *in vivo* [34]. Several fungal cell wall components, such as glucans and mannans, act as pathogen-associated molecular patterns (PAMPs). These components can be recognized by pattern recognition receptors (PRRs) of the innate immune system, like the TLR4 for *O*-linked mannans, the mannose receptor and Dectin-2 for N-linked mannans, and Dectin-1 for β-1,3-glucan (reviewed in [26]). On the other hand, although chitin recognition has been less studied, some work using shellfish chitin has suggested a recognition by C-type lectin receptors, such as the mannose receptor (MR), known to bind GlcNAc residues [12,35]. There are also reports suggesting an involvement of the TLR-2 and Dectin-1 receptors [13,15,24]. The few reports on the effects of chitin purified from *C. albicans* are variable in nature. One study showed that pretreatment of mice with chitin particles purified from *C. albicans* enhanced their survival after *C. albicans* infection and also enhanced the phagocytic and candidacidal activities of their peritoneal macrophages [19]. Another study showed that ultrapure chitin from *C. albicans* failed to induce any significant immune response when incubated with hPBMCs [29].

Previously reported methods for purifying chitin from *C. albicans* consisted of a step-by-step elimination of the outer components of the cell wall. An initial chemical treatment to eliminate the glycosylated proteins and some of the most soluble glucan fraction was achieved by boiling samples for 30 min in 5% KOH. After washing in de-ionized water, samples were autoclaved in a mix of glacial acetic acid and 40% hydrogen peroxide to weaken the bonds between the chitin and the glucans. The samples were then boiled three additional times in 5% KOH with washes in between. Finally the samples were washed thoroughly with de-ionized water until a white pellet was obtained and stored at −20 °C [29,30].

**Figure 3:**
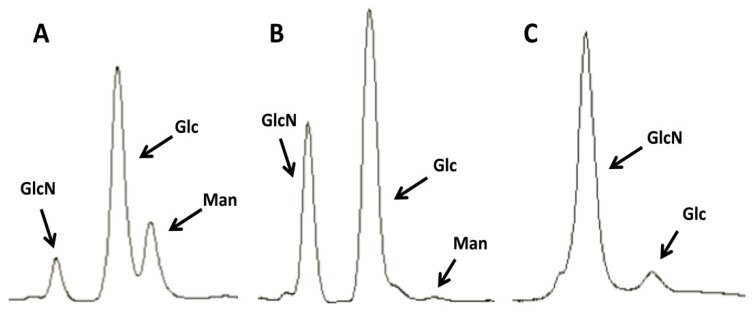
Purification of chitin from *C. albicans* yeast phase cells. Images representative of the HPLC profiles of *C. albicans* extracts at different stages of the chitin purification process. (**A**) Untreated cell walls; (**B**) after 3 KOH boils; (**C**) after an additional boil in a 1:1 mix of acetic acid and 40% H_2_O_2_. Man, mannose (mannans); Glc, glucose (glucans); GlcN, glucosamine (chitin).

Significant modifications to the above protocol were made to permit the elimination of the outer components of the cell wall with the use of simpler equipment. Unlike the commercial preparations of shellfish chitin, the *C. albicans* cell wall has a higher proportion of glycosylated proteins. The results showed that it takes up to three hot alkali boils to remove them completely (see Figure 3A,B). It was also found that it is very important to wash the pellets thoroughly, especially after the last boil, to obtain a dispersed sample that lacks clumps. Next, similar to the purification of commercial crab chitin described above, the use of an autoclave was eliminated (the fumes released during this step can corrode parts of the autoclaving apparatus, resulting in costly repairs). Instead, the sample was boiled once in the mix of acetic acid and hydrogen peroxide to eliminate most of the remaining glucan components of the cell wall by weakening their bonds with the chitin. Finally, after extensive washing the samples were stored at 4 °C because we observed undesired chitin aggregation when stored at −20 °C.

As seen in Figure 3C, the levels of glucans were greatly diminished after the acid boiling step, although most often it was difficult to eliminate the glucans completely, even after additional boiling in acid. Finally, the sample was washed at 2057 *×g* to both harvest a pellet enriched in bigger chitin particles and a supernatant enriched in SSCs, which was harvested by an additional centrifugation step at higher speed. This protocol is described more in detail in the Experimental Section*.*

**Figure 4:**
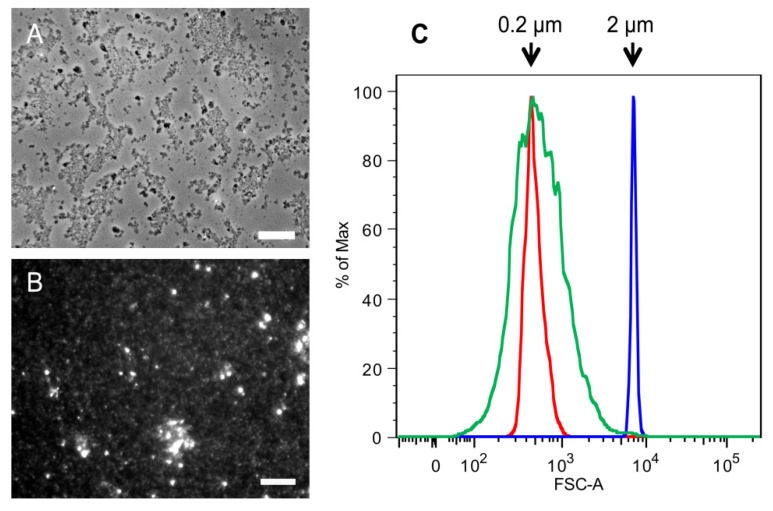
Comparison of SSCs and 2 k chitin particles from *C. albicans* yeast cells.Microscopic analysis of (**A**) 2 k chitin viewed by phase contrast microscopy; and (**B**) SSCs viewed by fluorescence microscopy after CFW staining; (**C**) Flow Cytometry analysis of SSCs. Red and blue indicate the profiles of 0.2 and 2 µm standard beads, respectively; green indicate the profile of SSCs. Bars, 10 µm.

Phase contrast microscopy was used to determine the size of the 2 k chitin particles and fluorescence microscopy and flow cytometry for the SSCs (Figure 4). Unlike the 2 k fraction of the commercial crab chitin, the *C. albicans* 2 k particles were small, very homogeneous in shape, and under the microscope we could see the formation of loose aggregates of diverse sizes (Figure 4A). Analysis of the *C. albicans* SSC particles showed that they were also highly homogeneous and had an average size of 0.2 µm (Figure 4B,C).

The different preparations of chitin purified from *C. albicans* cell walls were then tested for the ability to induce hPBMCs cytokines using ELISA, similar to the assays described above for the analysis of commercial crab chitin. Surprisingly, the immune profiles from *C. albicans* yeast chitin 2 k and SSC showed no significant induction of most cytokines after a considerable number of independent replicates (see Table 2), although the *p* values for IL-6 and TNFα almost reached significance for the SSC particles (*p* = 0.06 and 0.056 respectively). On the other hand, after 32 independent replicate analysis of the 2 k chitin, significant induction was only detected for the pro-inflammatory cytokine IL-1β. The 2 k fraction had aggregates of sizes that have been described by others as immunogenic. However, the fact that under the microscope the aggregates looked loose and very particulate could make inefficient their uptake by the immune cells. Phagocytosis of chitin particles is a requirement for their signaling capacity, as reported by others [11,12]. Indeed, use of the liposomal transfection reagent DOTAP to promote endosomal targeting of 2 k chitin particles from *C. albicans* enhanced dramatically the immune response (see Figure S2; note that the chitin used in those assays had been generated with an earlier version of the above chitin purification protocol that included extra alkaline boils after an acid treatment in an autoclave). This suggested both the presence of internal receptors for chitin and the need for chitin internalization to induce signaling. The reason for the likely failure of the *C. albicans* 2 k chitin to be taken up by the cells, but not the commercial crab chitin, could have to do with some of the physical properties in which they differed: the *C. albicans* 2 k chitin being small, granular and homogeneous while the 2 k fraction of commercial crab chitin was more heterogeneous in size and shape. Further evidence in support of this hypothesis is shown below.

**Table 2:** Cytokines after exposure of *C. albicans* yeast chitin to hPBMCs.

Cytokine		*C. albicans* SSCs (n = 22–28)	*C. albicans* 2 k chitin (n = 18–36)
IL-6	control	69 (57)	164 (142)
	chitin	267 (530)	379 (672)
	*p*	0.060	0.073
IL-1β	control	32 (21)	42 (6)
	chitin	37 (27)	187 (291)
	*p*	0.576	0.008
TNFα	control	34 (11)	110 (59)
	chitin	25 (15)	222 (246)
	*p*	0.056	0.076
IL-10	control	19 (26)	50 (76)
	chitin	21 (26)	63 (89)
	*p*	0.811	0.611

Chitin preparation (number of independent replicates); Mean (standard deviation); Values are given in pg/mL; Note that the number of replicates differs among cytokines: for SSCs: IL-6 and IL-1β, n = 28; IL-10, n = 26; TNFα, n = 22. For 2 k chitin: IL-1β and IL-10, n = 32; IL-6, n = 36; TNFα, n = 18.

### 2.4. Hyphal Chitin SSCs Behave Similar to C. albicans Yeast SSCs while the 2 k Fractions Show Differences Depending on Their Origin

Many animal and plant pathogenic fungi are dimorphic, being able to grow as budding cells and also as filaments (hyphae). The filamentous cells often have a slightly different cell wall composition. As an example, the cell walls of *C. albicans* hyphae contain less mannans and more chitin than in the round yeast cells (blastopores) [36]. These fungal filaments must be both flexible and rigid to penetrate tissues. In *C. albicans* the filaments acquire rigidity and stability at their tips through the covalent bonding of chitin and β-1,3-glucan by enzymes residing in the cell wall [37]. It was therefore possible that the chitin obtained from *C. albicans* hyphae had different properties than those observed above for chitin from *C. albicans* yeast cells. A similar hypothesis was addressed for hyphae belonging to two other opportunistic pathogenic fungi: *Aspergillus* *fumigatus* and *Mucor circinelloides*. Apart from the medical problems produced by mycotoxins or the invasive aspergillosis in immunocompromised patients, exposure to large amounts of *A. fumigatus* spores are known to cause allergies and chitin is known to act as an adjuvant in the process of *Aspergillus*-induced allergic sensitization [16]. The cell wall fragments containing covalently linked chitin and β-glucans also induced an allergic response in mice after intranasal administration [38]. *M. circinelloides* is also an opportunistic human pathogen that causes zygomycosis, rapidly invading blood vessels in debilitated patients, and its spores are known to activate human complement *in vitro* [39]. Supplementary Figure 1B–D show representative images of the filaments of these three fungal pathogens at the time of their harvest for the chitin purification procedure. Since there is scant information on the immune responses to the chitin belonging to the cell walls of these three organisms, one of the aims of this work was to contribute with new data on this matter.

**Figure 5:**
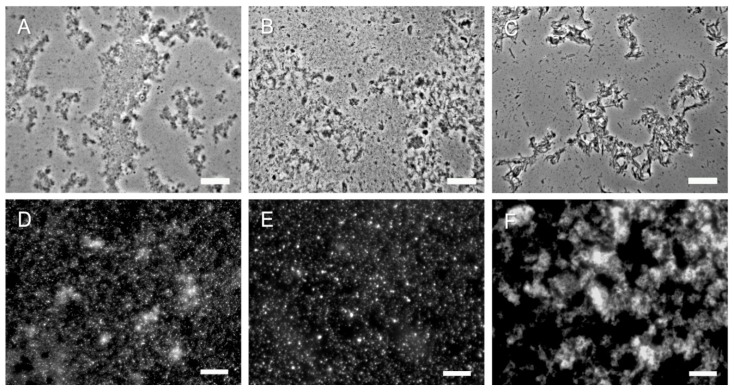
Representative microscopy images of SSCs and 2 k chitin from hyphae of three different fungi. (**A**–**C**) 2 k fractions from *C. albicans*, *A. fumigatus* and *M. circinelloides*, respectively (phase contrast); (**D**–**F**) SSCs from the corresponding fungi were stained with CFW and then viewed by fluorescence microscopy. Bars, 10 µm.

At the start of the purification process it was found that the harvest of fungal filamentous cells was easier by filtration than by centrifugation. In the latter case the pellets were not as compact as those originating from the round yeast cells and much material was lost if centrifuged. Following the purification protocol, all the hyphal SSCs looked quite similar by flow cytometry (data not shown) and by CFW staining and fluorescence microscopy (Figure 5D–F). The SSCs from *M. circinelloides* seemed to be more prone to clumping in loose aggregates (see Figure 5F). In addition, the 2 k chitin from hyphal *C. albicans* or *A. fumigatus* cells didn’t show any obvious difference from that purified from *C. albicans* yeast cells under the microscope (Figure 5A,B). On the other hand, when trying to isolate chitin from *M. circinelloides* following the same protocol, it was found to be more resilient to the alkali and acid treatments and complete breakdown of the filamentous structures was not achieved even after extra alkali or acid boils, despite producing similar HPLC profiles in terms of sugar composition in the end (data not shown). This made it more difficult to harvest SSCs from this species compared to the other two filamentous fungi.

Next, the immunogenic properties of the chitin purified from hyphal cells were analyzed. Since all of the SSCs harvested from these different filamentous fungi looked quite similar under the microscope, it was not surprising their shared lack of significant cytokine induction when co-incubated with hPBMCs for 24 h (see Table 3) and this was again in agreement with published literature [24].

Furthermore, co-incubation of 2 k chitin derived from *C. albicans* or *A. fumigatus* hyphae resulted in no detectable cytokine induction. The lack of effects for the *C. albicans* 2 k hyphal particles was surprising, since particles of yeast origin had previously resulted in the induction of IL-1β (see Table 2) and there were no obvious differences among the two sets of samples in their HPLC profiles or under the microscope. Interestingly, *M. circinelloides* 2 k chitin very significantly induced the secretion of the pro-inflammatory cytokines IL-6 and IL-1β by hPBMCs (Table 4).

**Table 3:** Cytokines produced after exposure of hyphal SSCs to hPBMCs. SSCs were purified from three different filamentous fungi as indicated.

Cytokine		*C. albicans* (n = 18)	*A. fumigatus* (n = 26)	*M. circinelloides* (n = 9)
IL-6	control	42 (32)	97 (84)	55 (75)
	chitin	56 (64)	370 (708)	46 (52)
	*p*	0.534	0.064	0.766
IL-1β	control	20 (0)	28 (25)	35 (25)
	chitin	24 (18)	35 (42)	27 (10)
	*p*	0.475	0.624	0.363
TNFα	control	36 (8)	37 (7)	41 (3)
	chitin	38 (11)	49 (36)	40 (0)
	*p*	0.583	0.116	0.332
IL-10	control	9 (2)	9 (1)	10 (3)
	chitin	8 (3)	10 (6)	11 (7)
	*p*	0.953	0.180	0.673

Chitin preparation (number of independent replicates); Mean (standard deviation); Values are given in pg/mL.

The above results portray clear differences in the signaling capacity of chitin from diverse origins. It is noteworthy that the 2 k chitin from both crab and from *M. circinelloides* had spike-like structures under the light microscope (see Figure 2A and Figure 5C), and also both induced the most cytokines. It is then possible that those structures were better recognized by the immune cells. On the other hand, the scaffolds generated by the 2 k chitin of *M. circinelloides* and some medium sized particles of the commercial crab chitin could be inducing a protective immune response due solely to their size.

**Table 4:** Cytokines produced by hPBMCs after exposure to 2 k chitin purified from hyphae*.*

Cytokine		*C. albicans* (n = 7)	*A. fumigatus* (n = 16)	*M. circinelloides* (n = 16)
IL-6	control	65 (55)	61 (49)	61 (49)
	chitin	394 (922)	105 (209)	1681 (1419)
	*p*	0.383	0.428	0.0004
IL-1β	control	40 (0)	40 (0)	40 (0)
	chitin	195 (410)	61 (58)	898 (1081)
	*p*	0.456	0.158	0.006
TNFα	control	41 (4)	50 (17)	50 (17)
	chitin	137 (254)	47 (14)	545 (1063)
	*p*	0.361	0.6	0.082
IL-10	control	12 (5)	16 (10)	16 (10)
	chitin	16 (14)	12 (8)	66 (120)
	*p*	0.475	0.469	0.119

Chitin preparation (number of independent replicates); Mean (standard deviation); Values are given in pg/mL.

In sum, the above results provide novel insights on the immune response to chitin from both commercial crab chitin and that of three different filamentous fungi. Furthermore, since the purification protocol was not as successful in breaking down the hyphal structures of *M. circinelloides* as for the other fungi, this has resulted in the suggestion that both the size and shape of different chitin architectures may influence their immune recognition in experiments using hPBMCs.

## 3. Experimental

### 3.1. Fungal Strains, Growth Conditions and Source of Commercial Crab Chitin

*Candida albicans* strain NGY152 [40]; *Aspergillus fumigatus* wild-type (237) [41]; *Mucor circinelloides* CBS277.49 [42]. *C. albicans* yeast cells were grown at 30 °C in YPD broth (1% (*w*/*v*) yeast extract, 2% (*w*/*v*) mycological peptone, 2% (*w*/*v*) glucose, supplemented with 80 μg mL^−1^ uridine). Filamentous cells were grown in RPMI-1640 medium (Sigma-Aldrich, St. Louis, MO, USA) in all cases, although for *A. fumigatus* and *M. circinelloides* higher yields can be achieved if grown in YPD or Sabouraud media. Fresh 5 mM GlcNAc was added to *C. albicans* cultures to achieve good filamentation. A temperature of 37 °C was applied for a minimum of 16 h to *C. albicans* and also to *A.fumigatus* as previously described [43], while *M. circinelloides* was grown at 30 °C. Filaments were harvested by filtration instead of centrifugation. The commercial crab chitin was obtained from Sigma-Aldrich (cat. # C-3387).

### 3.2. Determination of SSC Size by Flow Cytometry

Diverse chitin preparations (SSCs, harvested from the washes of the 2 k chitin fraction) were analyzed for size and shape by a BD Fortessa (BD Biosciences, San Jose, CA, USA) using as standards 0.2 µm red fluorescent FluoSpheres^®^ and non-fluorescent 2 µm and 4 µm polystyrene calibration beads (Life Technologies, Carlsbad, CA, USA). Concentrated chitin samples (~1 mg/mL) were stained in a 1:1 mix with 0.1% fresh and filtered CFW. Analysis was performed with FlowJo software version 7 (Tree Star, Ashland, OR, USA).

### 3.3. Purification of Chitin from Fungal Cells

Briefly, 500 mL of fungal cells were grown to saturation with shaking at 200 rpm. Absence of bacterial contamination was confirmed under the microscope. The cells were washed with de-ionized water until the supernatant was transparent. Cell pellets were suspended in 400 mL of 5% (*w*/*v*) KOH, and boiled up to three times for 1–2 h at 100 °C until no more color was produced. The preparation was then pelleted and washed at 2057 ×*g* for 10 min with de-ionized water until the supernatant was transparent. More washes took place after the last KOH boil, ensuring that the pellet would lose its stickiness and that the pH of the sample was neutral. Those last washes also required the use of a swinging bucket rotor and low brake settings to avoid losing sample material. The pellet was then suspended in 200 mL of 40% H_2_O_2_-glacial acetic acid (1:1) solution, and boiled for 2 h at 101 °C. Material was diluted with H_2_O and centrifuged at high speed. The supernatant was disposed of following local regulations and the pellet was washed multiple times with water for 20 min at 2057 ×*g*, also applying a low brake setting. That pellet, once reconstituted, was called 2 k chitin. The supernatants of those washes were later spun at 15,557 ×*g* (low brake settings) and the pellet containing super small chitin particles (SSCs) was suspended in a small volume of H_2_O (additional 2 k spins must be performed on the SSCs to get rid of carried-over particles). The sample was finally stored at 4 °C (aggregation of SSCs was observed when stored at −20 °C). To test for bacterial or yeast contamination aliquots of the sample were incubated in LB broth (1% (*w*/*v*) tryptone, 0.5% (*w*/*v*) yeast extract, 0.5% (*w*/*v*) NaCl) and YPD. To test for LPS contamination Limulus Amebocyte Lysate (LAL) kit QCL-1000^®^ (Lonza, Basel, Switzerland) was used. The degree of acetylation of the chitin samples was also measured as in [44], resulting in all the different chitin samples in this study being more than 90% acetylated. This protocol was applied to purify chitin from *C. albicans* and *A. fumigatus*, while in the case of *M. circinelloides* more boiling steps in acid were necessary to break down the filamentous structures.

### 3.4. HPLC Determination of Chitin Purity and Glucosamine Content

To determine the chitin purity in the preparations, samples were hydrolyzed for 3 h with 2 M trifluoroacetic acid at 100 °C. The acid was removed by evaporation at 65 °C, and the debris was suspended twice in 1 mL de-ionized water, which was evaporated off and finally suspended in de-ionized water. Samples of 20 µL were analyzed by high-performance anion-exchange chromatography with pulsed amperometric detection (HPAEC-PAD) in a carbohydrate analyzer system from Dionex and its Chromeleon software (Sunnyvale, CA, USA) as described previously [45]. High-purity monosaccharide standards used to generate pulsed amperometric response curves were from Sigma-Aldrich. To determine the glucosamine content in the samples they were hydrolyzed in 500 µL of 6 M HCl acid for 16 h at 100 °C. Evaporation of the acid took place at 80 °C, followed by at least two washes with 1 mL water, which evaporated off at 65 °C. Samples were finally suspended in a small volume of de-ionized water and aliquots were similarly analyzed by HPLC. Analysis of the glucosamine peaks helped determine the chitin content in the chitin batches, based on the peaks produced by known standards.

### 3.5. Cytokine Release by hPBMCs

hPBMCs and cytokine assays were performed as previously described [46]. Samples of blood extracted from the cubital veins of healthy volunteers were analyzed anonymously. hPBMCs (5 × 10^5^ cells in 100 µL) were incubated in round-bottom 96-well Nunclon plates (Nunc, Roskilde, Denmark) with 10 µg/mL of chitin samples in PBS. As controls PBS was added to the hPBMCs instead of chitin, and in all cases hPBMCs were treated in parallel sets with 1 µM latrunculin A (it disrupts microfilament organization) for 1 hour before the addition of the chitin: this was found very useful to discard those replicates whose signal persisted after latrunculin A treatment, because of the likelihood of having acquired at some point endotoxin contamination. After 24 h of incubation at 37 °C under 5% (*v*/*v*) CO_2_, the preparations were centrifuged and supernatants collected and stored at –20 °C until assayed. Cells were added 200 µL fresh medium and stored at −80 °C to measure non-secreted cytokines after three cycles of freezing/defrosting. All the cytokines were analyzed by enzyme-linked immunosorbent assays (ELISAs) using commercial Duoset kits from R&D Systems (Abingdon, United Kingdom). Optical densities were read in a microplate reader (VersaMax, Molecular Devices, Sunnyvale, CA, USA). The levels of non-secreted cytokines were found insignificant in our assays. For those assays utilizing the liposomal transfection reagent DOTAP (Roche, Basel, Switzerland) we pre-incubated concentrated chitin samples with 10 µg/mL DOTAP before diluting them for the incubations with hPBMCs. As control only DOTAP was used. The hPBMCs were also treated in parallel with 1 µM latrunculin A or 80 µM of the dynamin inhibitor Dynasore (Sigma-Aldrich) as controls, and in both cases the cytokine induction was abrogated. Independent replicates were obtained by incubating different chitin batches with hPBMCs from different donors. Although a minimum of 3 chitin batches was generated for each condition (commercial crab, *C. albicans* yeast and hyphae, *A. fumigatus* hyphae and *M. circinelloides* hyphae) the number of representative chitin batches used for the incubations with hPBMCs differed among conditions. Only those that had a similar appearance by microscopy, comparative purity by HPLC, and were free of endotoxin contamination were used. Numbers were as follows (chitin batches in parenthesis): yeast *C. albicans* SSCs (3), 2 k (9) and DOTAP (5); hyphal *C. albicans* SSCs (2) and 2 k (1); *A. fumigatus* SSCs (3) and 2 k (2); *M. circinelloides* SSCs (1) and 2 k (2); crab SSCs (2) and 2 k (1).

### 3.6. Statistical Analysis

Comparisons between values of untreated and chitin treated hPBMCs were performed for each cytokine using two-tailed student *t*-test on Excel. Values of *p* < 0.05 were considered significant.

## 4. Conclusions

Determining the response of the immune system to chitin is important, since it is a component of the cell walls of pathogenic fungi. Unfortunately, purifying chitin is difficult as it is not soluble and it is usually found crosslinked to other cell wall components that are difficult to eliminate. The difficulty in purifying chitin may underlie the conflicting data in the literature regarding the effects of chitin on the immune system. Therefore, this work aimed to improve previous chitin purification methods by examining the effects of additional hydrolysis steps to eliminate contaminants. Methods were also modified to avoid the use of special equipment or the need for an autoclaving step. The results show that additional hydrolysis steps can be used to more effectively eliminate contaminating glucans and mannoproteins from chitin obtained from different sources, including the cell walls of pathogenic fungi.

The chitin purified in this new way consisted of heterogeneous particles. Therefore, after the hydrolysis steps, the preparations were divided into two fractions by centrifugation. Larger chitin particles were obtained by centrifugation at 2,057 ×*g*, and termed 2 k chitin. The size range of these 2 k particles varied depending on the source of the chitin. The supernatants of the 2 k spins were collected and centrifuged at 15,557 ×*g*. Microscopic and flow cytometric analysis indicated that this fraction was composed of very small particles with a mean size of 0.2 µm, and hence they were named SSCs. These SSCs were very homogeneous across the preparations derived from different sources. Interestingly, the 2 k chitin and SSCs showed differential ability to stimulate hPBMCs to produce cytokines. The SSCs were unable to induce a significant immune response, in agreement with a previous report on the use of SSCs of crab origin [24]. In contrast, incubation of 2 k chitin with hPBMCs yielded variable results depending on the origin of the chitin. Potential reasons for this type of variability are discussed further below.

Initial experiments in this work were performed using commercial crab chitin that was subjected to additional alkali and acidic treatment steps. This reduced the amounts of glucan contaminants, although they were not completely removed. The results of the immune assays with crab chitin showed a clear induction of the pro-inflammatory cytokines IL-6, IL-1β and TNFα. In addition, there was no significant induction of the anti-inflammatory cytokine IL-10. Previous work by others has shown contradictory effects of chitin on the immune system when using shellfish chitin. While most studies showed a pro-inflammatory effect [6,7,8,11,12,13,14,8,11], a few publications have reported a significant induction of the immunosuppressing cytokine IL-10. In two such studies particles of shellfish origin in the size range of 2–10 µm induced both *in vivo* and *in vitro* the secretion of IL-10 by murine cells [23,24]. The 2 k chitin from crab origin in the present study contained particles of similar sizes as those described above as being either pro-inflammatory or anti-inflammatory. Unfortunately, the many differences in the ways the experiments were carried out in the different studies makes them difficult to compare. Among the possible reasons that could account for the different results may be the use of different chitin concentrations in the *in vitro* assays, the different cell types used, or the extra chemical treatments used in this study to purify commercial crab chitin. Thus, although the results of this study indicate a pro-inflammatory response to crab chitin, there is not yet a clear understanding of why results differ so much between different labs, and further studies with standardized procedures are needed to resolve the current debate.

In contrast to crab chitin, the experiments using 2 k chitin purified from hyphal cells of *C. albicans* and *A. fumigatus* failed to induce cytokine responses (Table 4). The results for *C. albicans* hyphal 2 k chitin are in agreement with a previous study showing that *C. albicans* chitin purified with a protocol that included autoclaving in acid followed by three alkaline boils and then sedimentation at 2057 ×*g* didn’t induce detectable responses when similarly co-incubated with hPBMCs, even at concentrations 20 times higher than those in the present study [29]. The failure of hyphal chitin from *A. fumigatus* to induce detectable cytokine responses is in agreement with a previous study which found that only a small effect was induced by particles of either chitin or β-glucan alone, while a pro-inflammatory immune response was significantly higher when chitin and β-glucan were crosslinked together, both *in vivo* and *in vitro* [38]. Further comparisons are limited, since there are few previous reports on the immune response to *A. fumigatus* chitin, as most previous studies on cell walls or chitin obtained from *Aspergillus spp*. have focused on allergic responses [16,20,47]. Nonetheless, the present study provides an important basis of comparison for future studies.

Some types of fungal chitin were able to induce cytokine responses, as was seen for 2 k chitin purified from *C. albicans* yeast cells and 2 k chitin of *M. circinelloides.* The 2 k chitin purified from *C. albicans* yeast cells weakly induced the secretion of the pro-inflammatory cytokine IL-1β, whereas the hyphal 2 k chitin of *M. circinelloides* strongly induced the pro-inflammatory cytokines IL-6 and IL-1β. (Table 2 and Table 4). The *C. albicans* 2 k chitin particles from yeast cells appeared visually similar to the hyphal 2 k chitin, so it is not clear why it was able to weakly induce IL-1β. In contrast, the stronger induction of cytokines by *M. circinelloides* 2 k chitin correlated with this type of preparation having a very different morphology under the microscope compared with the chitin of the other fungi. It looked as a disorganized array of broken filaments and spikes despite having been subjected to more chemical treatments than the preparations from the other two fungi. The more heterogeneous nature of the *M. circinelloides* 2 k chitin was similar to 2 k chitin from commercial crab shells. Both types of 2 k preparations were not only more heterogeneous in size, they also contained particles of different shapes, including spike-like structures. Since the 2 k chitin from commercial crab shells and *M. circinelloides* were also the most effective at inducing cytokine responses, this suggests that the shape of the chitin particles, as well as the size, could be contributing to immune recognition. Further studies will be required to define how the shape of the chitin particles affects their ability to induce an immune response. An interesting approach would be to analyze chitin from the same source and degree of purity that has been engineered for form diverse shapes.

One other factor that could help explain the variable immune response to chitin of diverse origins is the effect of the chemical and physical treatments on the structure of the chitin. There is the possibility that the different types of harsh treatments used to purify chitin may alter its ability to be recognized by the immune cells. Commercial shellfish chitin is typically generated by industrial processes that include chemical treatments comparable to those used in the present study. One possible approach to address this question in future studies would be to carry out more detailed physical and chemical analyses on the structure of the chitin after the different purification approaches to help reach an understanding on why there is so much variability in the published reports on the immune responses to chitin. As an example, infrared spectroscopy and X-ray diffraction can be used to better measure the degree of acetylation of the chitin samples and the differences in their ultraestructure, respectively, as in previous studies [48]. Another way to examine this would be to try to avoid harsh treatments by using gentler biological purification methods [28]. An alternative type of approach would be to avoid purification methods and to examine the response to chitin in whole fungi. Although it is highly desirable to use purified chitin as a way to more specifically define the immune response to this component, the immune system is unlikely to be exposed to purified chitin during fungal infections. Chitin in the cell walls of fungi is covalently attached to glucans, which by itself could create a novel PAMP. Thus, future studies could be performed to understand how the cell wall is sequentially digested inside the phagolysosomes to observe whether there are differences in the types of cytokines released at different stages of degradation of the cellular material. This could be analyzed by making use of both reporter genes for cytokines and fluorescence microscopy on stained fungal cell walls.

In summary, this work has shown an improved method for purifying chitin from the cell walls of common fungal pathogens and commercial crab shells. The chitin produced was either inert or induced a pro-inflammatory immune response. These results suggest that not only the size of the chitin particles but also their shape, may be predictors of their immunogenicity. Furthermore, we have proposed experiments to gain insights on the issue of why there are conflicting reports in the literature regarding the immune response to chitin particles of both shellfish and fungal origin.

## Abbreviations

SSCs: super small chitin particles
hPBMCs: human peripheral blood mononuclear cells
GlcNAc: *N*-acetylglucosamine
GlcN: glucosamine
CFW: calcofluor white

## Supplementary Materials

Supplementary materials can be accessed at:  http://www.mdpi.com/1420-3049/19/4/4433/s1.
