# Supplementary material for: The Effect of Chitin Size, Shape, Source and Purification Method on Immune Recognition
Source: Molecules. 2014 Apr 10;19(4):4433–51. doi: 10.3390/molecules19044433 (PMC6271096; doi:10.3390/molecules19044433)
Supplement: Supplementary file 1 [file molecules-19-04433-s001.pdf]

Supplementary Materials

Figure S1. Representative images of the different fungal cultures at the time of harvest. (**A**) *C. albicans* yeast cells; (**B**) *C. albicans* hyphae; (**C**) *A. fumigatus* hyphae; (**D**) *M. circinelloides* hyphae. Bars, 50 μm.

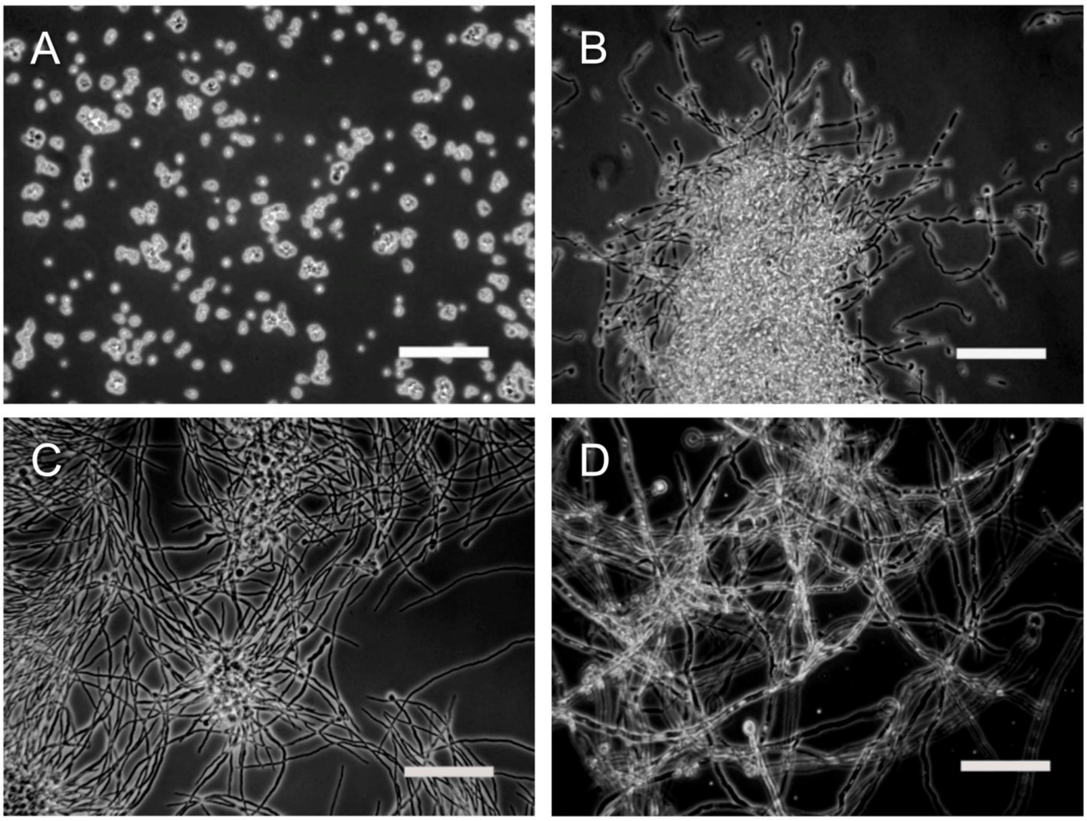

Figure S2. *Endosomal transfection of 2 k chitin particles*. The incubation of 10 μg/mL chitin particles with 10 μg/mL DOTAP significantly increases the induction of pro-inflammatory cytokines. As a control, incubation of PBMCs with 1 μM latrunculin A for 1 h before exposure to chitin blocks the uptake. (**A**) IL-6; (**B**) IL-1β; (**C**) IL-10.

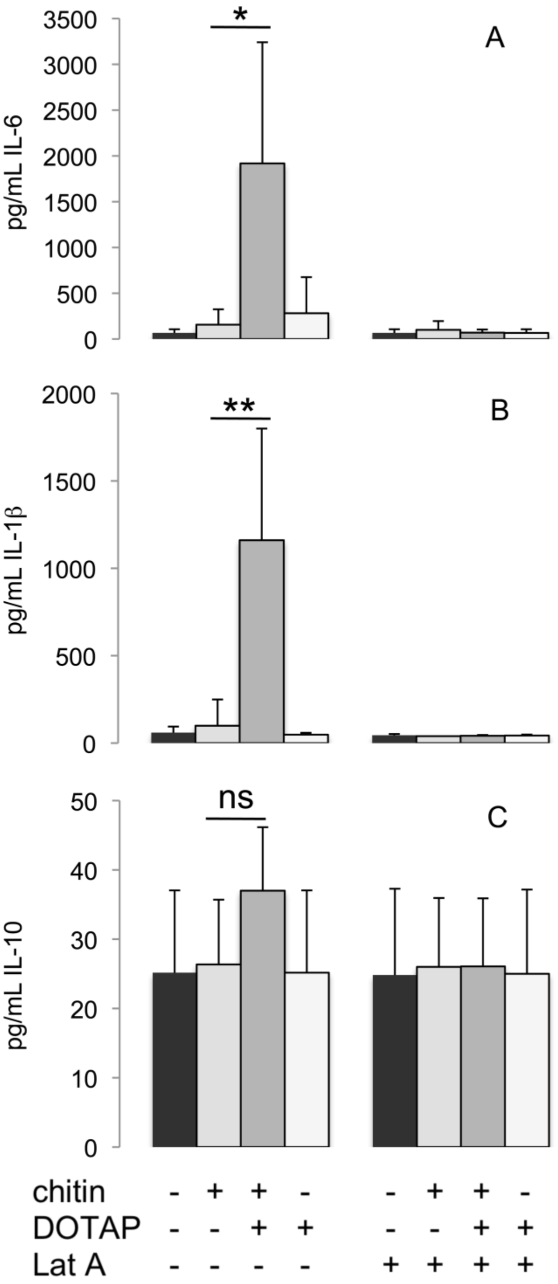

Note: The 2 k chitin samples used for this experiment had been obtained using a more primitive protocol that included an autoclaving step in acid followed by KOH boils. * $p < 0.05$; ** $p < 0.001$; ns = not significant.
